# Smart Magnetic Drug Delivery Systems for the Treatment of Cancer

**DOI:** 10.3390/nano13050876

**Published:** 2023-02-26

**Authors:** Angela Spoială, Cornelia-Ioana Ilie, Ludmila Motelica, Denisa Ficai, Augustin Semenescu, Ovidiu-Cristian Oprea, Anton Ficai

**Affiliations:** 1 Department of Science and Engineering of Oxide Materials and Nanomaterials, Faculty of Chemical Engineering and Biotechnologies, University Politehnica of Bucharest, 1-7 Gh Polizu Street, 011061 Bucharest, Romania; 2 National Centre for Micro and Nanomaterials, and National Centre for Food Safety, Faculty of Chemical Engineering and Biotechnologies, University Politehnica of Bucharest, 313 Spl. Independentei, 060042 Bucharest, Romania; 3 Department of Inorganic Chemistry, Physical Chemistry and Electrochemistry, Faculty of Chemical Engineering and Biotechnologies, University Politehnica of Bucharest, 1-7 Gh Polizu Street, 050054 Bucharest, Romania; 4 Departament of Engineering and Management for Transports, Faculty of Transports, University Politehnica of Bucharest, 313 Spl. Independentei, 060042 Bucharest, Romania; 5 Academy of Romanian Scientists, 3 Street Ilfov, 050045 Bucharest, Romania

**Keywords:** magnetic nanoparticles, targeting nanoparticles, linkers, passive targeting, active targeting

## Abstract

Cancer remains the most devastating disease, being one of the main factors of death and morbidity worldwide since ancient times. Although early diagnosis and treatment represent the correct approach in the fight against cancer, traditional therapies, such as chemotherapy, radiotherapy, targeted therapy, and immunotherapy, have some limitations (lack of specificity, cytotoxicity, and multidrug resistance). These limitations represent a continuous challenge for determining optimal therapies for the diagnosis and treatment of cancer. Cancer diagnosis and treatment have seen significant achievements with the advent of nanotechnology and a wide range of nanoparticles. Due to their special advantages, such as low toxicity, high stability, good permeability, biocompatibility, improved retention effect, and precise targeting, nanoparticles with sizes ranging from 1 nm to 100 nm have been successfully used in cancer diagnosis and treatment by solving the limitations of conventional cancer treatment, but also overcoming multidrug resistance. Additionally, choosing the best cancer diagnosis, treatment, and management is extremely important. The use of nanotechnology and magnetic nanoparticles (MNPs) represents an effective alternative in the simultaneous diagnosis and treatment of cancer using nano-theranostic particles that facilitate early-stage detection and selective destruction of cancer cells. The specific properties, such as the control of the dimensions and the specific surface through the judicious choice of synthesis methods, and the possibility of targeting the target organ by applying an internal magnetic field, make these nanoparticles effective alternatives for the diagnosis and treatment of cancer. This review discusses the use of MNPs in cancer diagnosis and treatment and provides future perspectives in the field.

## 1. Introduction

Cancer the second most widespread disease as 14.6% of all human deaths are a consequence of cancer. According to the American Cancer Society, worldwide cancer represents one of the major public health problems, currently surpassed only by cardiovascular diseases. The International Agency for Research on Cancer (IARC) has estimated that without increased global investment in cancer research and the application of existing knowledge on cancer control, and without significant efforts to improve global cancer control, cancer deaths could increase to 12.9 million/year by 2030 [1].

Cancer is a disease characterized by uncontrolled, random, invasive cell division. Over the years, special efforts have focused on detecting cancer risk factors. For some types of cancer, the aetiology has been associated primarily with the specific environment, such as radiation and pollution, but also with an unhealthy lifestyle, such as a poorly balanced diet, smoking, stress, and lack of physical activity. All these factors strongly influence the development of different forms of cancer [2,3]. Inherited genetics is another determining factor in cancer occurrence with 5–10% of cases being due to this factor [4]. Advancing age is another crucial risk factor for cancer, and many individual cancers have an increased mutation risk and aggregation of factors connected with age [5].

Cancer treatment includes surgery, radiation therapy, immunotherapy, and chemotherapy. Chemotherapy is used in over 50% of cancer cases as standard treatment, including metastatic cancers [6]. A major drawback of chemotherapy involves the reduced effectiveness of targeted drug delivery to tumour cells, causing unintended penetration of drugs into healthy cells and tissues. They could lead to side effects at a systemic level, including fatigue, hair loss, nausea, vomiting, and increased infections due to low blood cell counts (by affecting the blood-forming cells of the bone marrow). Higher doses of anticancer drugs are used to achieve the necessary drug concentration in the tumour cells, causing even more side effects due to the toxicity of the chemotherapeutic agents on healthy cells and tissues [7]. Another significant disadvantage of chemotherapy is a tumour’s intrinsic or acquired resistance to the drug, which often leads to disease reoccurrence and further decreases therapeutic outcomes. During the last decades, treatment resistance has been a major research topic leading to discoveries, such as cancer stem cells, sequence mutations, and bidirectional inter-conversion of cancer stem and non-stem cell populations [8].

Nevertheless, implementing successful cancer treatment protocols that lead to very good outcomes will require surpassing these elements of difficulty by a considerable refinement of our knowledge concerning the treatment, and therefore improving the survival chances of the patients. The current anticancer drugs are very potent killing factors. The next logical step is to manage, and design targeted delivery systems, which can release the drug inside the tumour, blocking the drug’s capability to attack the healthy tissue [9]. Such results can be obtained by magnetic carriers, which can be designed to simultaneously assure targeting and triggering, and thus to develop smart systems able to generate personalized therapy.

## 2. Nanostructured Carriers

In an attempt to replace current cancer treatments, various nanomaterials, such as liposomes [10,11,12], immunoliposomes [13], MNPs [14,15,16], polymers, nanogels, etc., are being used in clinical trials to ensure targeted delivery of the biologically active agents into the desired tissue/organ, according to a desired release profile. Table 1 illustrates the most important nanocarriers in the targeted delivery of different chemotherapeutic agents. 

Such carriers increase the circulation time in chemotherapeutic agents’ bloodstream, improving their accumulation and retention in tumour cells or tissues. It sometimes increases chemotherapeutic agents’ release across physiological barriers at the disease site [17,18].

**Table 1:** Type of nanoparticles used as carriers.

Type of Carriers		Advantage	Disadvantage	References
Inorganic carriers	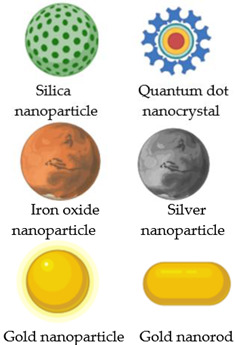	Small size; Special magnetic, electric, and optic properties; Tuneable size, structure, and functionalization; Suitable for theranostic application.	Low solubility; Toxicity.	[19,20]
Lipid-based carriers	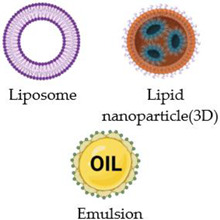	Ease formulation for specific applications; High bioavailability; Hydrophilic and lipophilic carriers; Chemical modification; Improve blood circulation.	Carrier flexibility; Low encapsulation efficiency.	[19,20]
Polymeric carriers	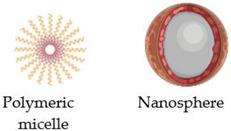	Easily controlled; Surface modification; Biodegradable; Hydrophilic and hydrophobic carriers.	Self-aggregation; Toxicity.	[19,20]

As a result of their nanometric dimensions (10 to 100 nm) and the enhanced permeability and retention rate (EPR) of the tumoral cells, these nanocarriers accumulate in tumour cells or tissues much faster than in healthy cells or tissues. This phenomenon can be explained by the fact that the tumour cells are more active, and internalization is also faster because they need more nutrients and oxygen to grow (which is why angiogenesis is faster), leading to increased therapeutic efficacy and reduced side effects [21]. The carrier’s size is essential because small particles, such as the quantum dots, facilitate cell internalisation, and the surface charge and chemistry are crucial [22].

Among the various MNPs used as systems for releasing chemotherapeutic agents, special attention has been shown to Fe_3_O_4_ nanoparticles. They offer opportunities for biomedical applications due to their superparamagnetism [23]. However, there are a series of major disadvantages, such as high susceptibility to acid and oxidative degradation. Additionally, the high degree of agglomeration is due to strong van der Waals and magnetic attractions between particles, which cause the accumulation of MNPs and limit the practical applications of these nanoparticles. To overcome these disadvantages, coating the nanoparticles with an outer protective layer is an effective strategy to maintain the magnetic components’ stability and avoid excessive agglomeration [24,25]. An efficient and often used procedure to achieve this is by encapsulating Fe_3_O_4_ nanoparticles in an inorganic (C, SiO_2_, ZnO, etc.) or organic coating (PEG is the most used polymer) to obtain magnetic systems with a core–shell structure [26,27], which can expand their technical application as a result of the unique characteristics of the coating (high stability in biological conditions) and their ability to provide a platform for chelating groups. However, the use of Fe_3_O_4_@inorganic for cancer treatment is limited. Most inorganic shells are hydrophobic and chemically inert, which disadvantages their applications in an aqueous environment. Indeed, additional surface modification can be done to achieve the proper stability, hydrophilic/hydrophobic ratio, bioaccumulation tendency, and release rate, but also an appropriate reactivity of the surface [28].

Therefore, an organic coating of MNPs can accomplish more than one goal: provide a suitable surface for hydrophilic interactions, loading capacity similar to a sponge, antibody functionalization for targeted delivery, biocompatibility to evade the human body immune system, and capacity to deceive the tumour cell defence mechanisms [29,30,31,32]. A promising synthetic material extensively reported in the literature for surface modifications of MNPs is polyethylene glycol (PEG) [33], a hydrophilic, highly water soluble, biocompatible, non-antigenic, and protein-resistant polymer [24]. According to Tai et al. [34], compared to unmodified MNPs, PEG-coated MNPs showed high colloidal stability for up to 21 days. This long-term stability is not always necessary, as cytostatic release occurs within only a few days. Depending on the application, release profile, loading capacity, and targeted delivery are much more important. Therefore, finding suitable capping agents for a particular application is still a limitation/challenge of current approaches. 

In oncology, MNPs are specially designed as Trojan horses. They are expected to internalize into the tumoral cells, followed by additional therapies, such as the release of antitumoral agents or radiotherapy [35,36,37]. This approach is beneficial because, in this way, the systemic toxicity is decreased (the targeting being assured by magnetic fields or by decorating these carriers with specific receptors: folic acid, specific peptides, and antibodies being some of the most studied). These carriers primarily accumulate in the desired cells and tissues [38,39,40].

## 3. Antitumoral Agents and Nanomedicine

The National Institute of Health has defined “nanomedicine” as the applications of nanotechnology for the treatment, diagnosis, monitoring, and controlling of biological systems. Scientists have focused on researching the adequate modality to deliver and target pharmaceutical, therapeutic, and diagnostic agents, and they turned towards nanomedicine. Using nanomedicine in cancer means identifying a precise target with specific clinical conditions and choosing suitable nanosystems-drug conjugates to achieve the desired response while minimizing the side effects of anticancer drugs. Therefore, today’s nanotechnology and nanomedicine approaches have designed and expanded the basis of drug formulations for humankind’s benefit [41].

Nanomedicine has great potential to improve anticancer therapy. Thus, a relatively small number of nanomedicine products are approved for clinical trials that could ensure specific therapeutic benefits for patients. Still, the perspectives in the field are immense. Improving nanomedicine’s clinical impact in cancer therapy requires novel perspectives on establishing smart strategies. By integrating clinical trials, pharmaceutical companies, and the authorities in developing innovative anticancer drugs, some crucial achievements are expected, including improved efficiency and lower toxicity [42,43]. Nanoscience has shown that designing various drug formulations with enhanced diagnostic and therapeutic effects could shift into synthesizing a mono-nano-drug [44,45]. Several features must be followed to configure nano-drug formulations. For example, their core is based on organic or inorganic molecules, or a combination must hinge for the intended use. Properties, such as surface charge, tuneable size, and hydrophobicity, could be improved for the desired function [46,47]. In this viewpoint, nanomedicine shifted attention to a significant class of nanoparticles, such as MNPs, with great applicability in developing platforms to combat cancer [48].

MNPs have unique characteristics, being able to be used successfully in diagnosing and treating cancer due to their special physicochemical properties. Due to their easy synthesis, high affinity to surface functionalization, low toxicity, and good biodegradability, MNPs act as outstanding imaging tools and drug delivery carriers in cancer therapies [49,50,51]. MNPs possess great biomedical potentials, such as biosensing [52], magnetic hyperthermia [53], MRI [54], and controlled drug release [55]. Due to their high magnetization, MNPs have drawn attention towards a new imaging technique. 

**Figure 1:**
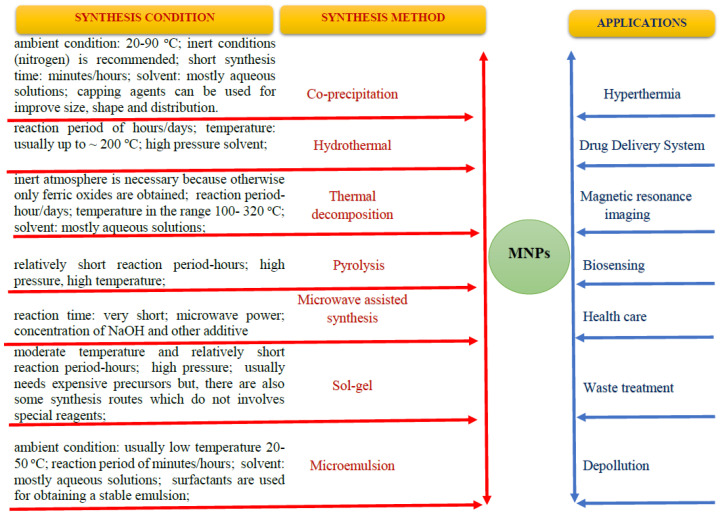
Correlation between synthesis method and application.

Moreover, to understand the applications of MNPs in cancer therapy, one must consider their synthesis, characterization, size, shape, and coating (Figure 1). MNPs usually have a magnetic core–shell and a polymeric coating, showing that essential properties are enhanced through functionalization, decoration, and surface coating. The literature includes some traditional syntheses for MNPs, such as co-precipitation, sonochemistry, hydrothermal, or solvothermal methods, reverse microemulsion, pyrolysis, and thermal decomposition [56].

Lately, researchers have designed novel synthesis strategies, such as biogenic and microfluidic synthesis. Microfluidic synthesis uses many materials, such as glass, silicon, ceramics, and polymers, which provide significant advantages over the final size, shape, and homogeneity of the formed nanoparticles. For example, Cabrera et al. [57] designed a latex-based microfluidic platform synthesizing gold and iron oxide nanoparticles. It is worth mentioning that the obtained nanoparticles could be mixed and form 10 nm-sized iron oxide NPs decorated with 4 nm AuNPs with monodisperse core sizes.

This new application of MNPs implies the ability of the nanoparticles to detect cancerous cells within diverse necrotic tissues. This capacity could be explained by the fact that the shape, and physical and chemical properties of MNPs are interdependent. The main condition-based advantages of the MNPs are morphology, chemical composition, shape, size, magnetic and functionalization features, which are very important aspects for the desired biomedical usage [58,59]. The magnetic properties of MNPs could be customized with a biocompatible coating to increase their specificity for targeted cancerous tissue. The biocompatible coating can form many surface modifications that could transform into various multi-functionalities approaches of MNPs [60].

Nanoparticles synthesized in the presence of dispersing agents are more reactive than materials synthesized in the absence of dispersing agents due to their high surface/volume ratio [56]. Additionally, they show a high degree of protection against oxidation due to MNPs coating. By covering the MNPs, the degree of toxicity in the body is prevented and reduced in the case of their use in in vivo applications. The choice of the coating materials has to take into account the nature of the coating, especially from the point of view of stability, and the possibility of further physical or chemical functionalization, according to the desired final application. The following examples show a range of coating materials used to develop medical application carriers [61].

The functionalization of the surfaces of magnetic materials using organic linkers is widely used because organic linkers confer specific surface properties to the various biomolecules to be linked. In the specialized literature, a series of organic, such as amines, carboxylic acids, aldehydes, and thiols linkers, are used in the functionalization process of MNPs, the most organic linkers used are those that create electrostatic interactions. This is because the binding strength is relatively easy to manipulate, either by adding ions or changing the pH of the medium. For example, for the use of MNPs as carriers for gene delivery, the surface of the MNPs must be strongly positively charged, as they must ideally bind through electrostatic interactions, a large amount of negatively charged DNA molecules. These electrostatic interactions allow the release of genes after the internalization of MNPs in the cell [62]. For the delivery of drugs, such as ibuprofen and aspirin, which have negatively charged groups (e.g., carboxylate, sulphonate, etc.) in their molecular structure, the surface of MNPs must be functionalized with organic linkers that decorate the surface of these nanoparticles with positive groups, such as ammonium salts. Once these MNP-drug systems reach the target organs, drug delivery will be triggered due to the anion exchange (chlorides and phosphates) [61].

Another material used to modify the surface of MNPs is amorphous silica and/or mesoporous silica. The modification of MNPs with silica is usually carried out as the hydrolysis of tetraethyl orthosilicate (TEOS) at a neutral-slightly alkaline pH by salinization with functionalized silanes such as 3-aminopropyl trimethoxysilan or the neutralization of silicic acid.

Using natural polymers in coating MNPs is a common alternative, due to their high biocompatibility, to use them in medical applications. For example, MNPs covered with dextran are used in the treatment of cancer, the modification of the surfaces of the MNPs being possible by making hydrogen bonds between the -OH groups in the structure and those behind the surface of the MNPs. Dextran was used with other organic (alginate, chitosan, poly-L-lactic acid) and inorganic (silica) polymers to improve the surface properties and generate new ones [61].

The following section of the review is devoted to using superparamagnetic iron oxide materials (SPIONs) in cancer therapy. SPIONs are considered promising nanostructured platforms suitable for drug delivery due to their functionalization ability, good biocompatibility, and enhanced contrast effects for MRI [63]. 

Zuvin et al. [64] showed the anticancer properties of SPIONs stabilized with polyacrylic acid on breast cancer tumours. The modified NPs successfully provided more effective treatment, showing low toxicity, high stability, and ultra-small particle size. 

A study by Kandasamy et al. [65] reported designing a new multifunctional magnetic-polymeric NPs built from ferrofluids by encapsulating hydrophobic SPIONs stabilized with oleylamine into the PLGA-based NPs, with two drugs, curcumin or verapamil. The authors investigated the biocompatibility, magnetic properties, and heating capacity of the formed MF-MPNs. The results showed that PLGA encapsulation significantly improved the stability of SPIONs with minimal toxicity and maximum treatment efficiency.

In this case, polycaprolactone (PCL)-coated SPIONs were used to design a new therapeutic drug with improved thermosensitivity and cytocompatibility. The obtained nanomedicine showed significant advantages, high stability, good dispersion, cytocompatibility, and the possibility of high control when heating [66].

## 4. Magnetic Hyperthermia 

Magnetic hyperthermia is another treatment option, along with chemotherapy and radiation therapy, used in cancer therapy [67]. For therapeutic purposes, hyperthermia therapy (HT) involves exposure to high temperatures of the whole body or specific areas of the body to achieve a therapeutic effect. In recent years, this therapy has begun to be increasingly used as a complementary form of cancer treatment [68].

Techniques currently used to achieve a localized hyperthermic effect are radiofrequency, ultrasound, microwave, laser, and MNPs. The use of MNPs to generate therapeutic hyperthermia is known as magnetic hyperthermia (MHT), and was used as a cancer therapy in 1957 [68]. MHT was first used when Gilchrist et al. [69] selectively heated tumours using magnetic particles by applying an alternating magnetic field (AMF). Later, MHT was introduced clinically as an alternative approach for the local treatment of tumours without affecting the surrounding healthy tissues [70,71,72,73].

Hyperthermia treatment generally uses heat from various sources, such as electromagnetic waves or ultrasound, to destroy cancer cells by denaturing the proteins that combine the cell membrane and cytoplasm. Techniques currently used to achieve a localized hyperthermic effect are radiofrequency, ultrasound, microwave, laser, and MNPs. The idea of hyperthermia used as an artificial temperature inducer above the threshold of 46 °C in the human body has been around for decades. Hyperthermia is usually used with other therapies, such as chemotherapy or radiotherapy [5,35,36,37].

Available MHT techniques do not efficiently direct the heat to the tumour, thus exhibiting low efficiency. Consequently, this lack of efficiency has led to the development of nanomaterials with properties capable of dissipating energy at the tumour site, increasing efficiency and specificity for deeper tumours [6,21]. In addition, the development of diverse strategies for the cellular internalization process and/or paramagnetic nanoparticles [22], such as iron oxide nanoparticles, contributes to increasing the efficiency of this therapy.

Heat destroys tumour cells due to the low dissipation of thermal energy associated with the heterogeneity of oxygen intake and nutrient demand caused by the excessive tortuous branching of blood vessels and the absence of lymphatic vessels. The increase in temperature leads to the modification of the functions of structural and enzymatic proteins in tumour cells, followed by the modification of the cell proliferation index that finally shows their apoptosis/necrosis [74,75].

The heating capacity is closely related to the NPM material’s properties and the parameters of the applied field. However, for nanostructured magnetic materials, the heating effectiveness relies on the correlation between the intrinsic time-dependent relaxation processes of the NPM magnetic moments and the time scale of the AMF field vector [76].

The MHT technique is based on two MNP relaxation processes, Néel relaxation and Brownian relaxation, both associated with intracellular and extracellular MHT processes [77]. In the case of nanoparticles internalized in tumour cells, by applying an alternating magnetic field (AMF), only the Neel relaxation contributes to the thermal energy (intracellular MHT). Brownian relaxation does not contribute to the thermal energy due to the high viscosity of the medium, which does not allow the nanoparticles to rotate freely.

For nanoparticles not internalized in tumour cells, the contribution of Néel relaxation and the contribution of Brownian relaxation are relevant to the extracellular MHT process [78].

**Figure 2:**
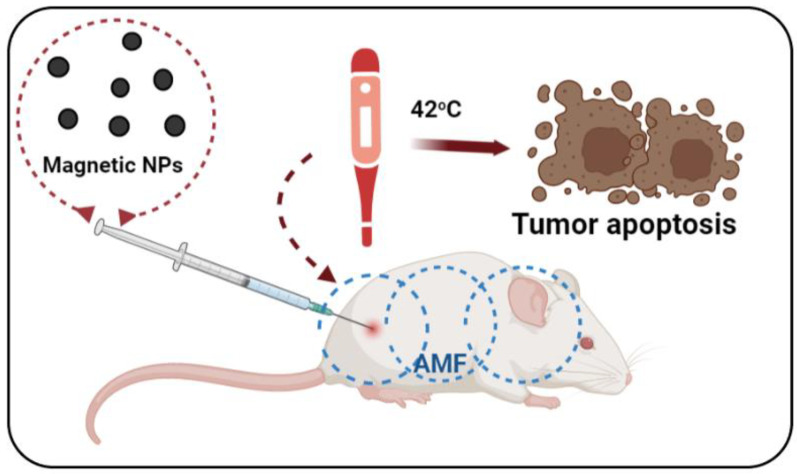
Schematic illustration for the MNPs-mediated MH working mechanisms under AMF.

MHT consists of using the heat generated by MNPs when applied to an alternating magnetic field (AMF) to destroy cancer cells by denaturing the proteins that make up the cell membrane and cytoplasm, resulting in altered physiology of the cancer cell, which ultimately leads to their apoptosis/necrosis (Figure 2).

In exposing iron oxide nanoparticles to alternating magnetic fields, heat is generated by Neel relaxation and Brownian rotation. Ideally, using active or passive targeting processes, MNPs can be targeted to the tumour, where they accumulate. Thus, by applying an external magnetic field, the temperature of the cells around the MNPs is considerably increased compared to the temperature of the cells further away. Thus, cells in the vicinity of MNPs can be selectively destroyed, at the same time reducing the body’s exposure period to external stimuli (Figure 2).

MNPs-mediated MH therapeutic treatment (MNPs-MH) helps achieve intracellular hyperthermia [72] by applying an alternating magnetic field. This directly leads to the therapeutic heating of cancer cells, the local and homogeneous heat obtained leads to higher selectivity and efficacy of therapy. The most significant advantage of MNPs-mediated MH (MNPs-MH) is deep tissue penetration and selective killing of cancer cells without affecting healthy cells and tissues. As a result of these advantages, MNPs-MH-based tumour treatment was introduced into clinical trials not long ago, and has been successfully used for the treatment of glioblastoma, pancreatic cancer, and prostate cancer [79]. This technique has proven effective, producing good results in various preclinical studies performed on animals and phase III clinical studies on humans [80,81].

## 5. Passive Targeting

Passive targeting of a target organ is an essential process because the free accumulation of MNPs in the tumour area leads. In some cases, effective MRI detection facilitates cancer diagnosis and, implicitly, its subsequent treatment. Nanoparticles preferentially accumulate in tumour tissues due to EPR for suitable-sized NPs [82,83,84]. It has been demonstrated that an NP with d = 10–100 nm may be accumulated predominantly in tumours, compared to normal tissues. Passive targeting is strongly influenced by the nanoparticles’ size, surface charge, and hydrophobicity [85]. For example, NPs smaller than 20 nm predominantly accumulate in the kidneys. In comparison, the NPs with d = 30–150 nm predominantly accumulate in the bone marrow, heart, kidneys, and stomach. NPs with d > 150 tend to bio-accumulate in the liver and spleen. Another important property that influences passive targeting is represented by retention times. Due to opsonisation, Gobbo et al. [86] showed that hydrophobic and positively charged structures have short circulation times. In contrast, hydrophilic and negatively charged nanostructures have long circulation times [86,87]. 

**Figure 3:**
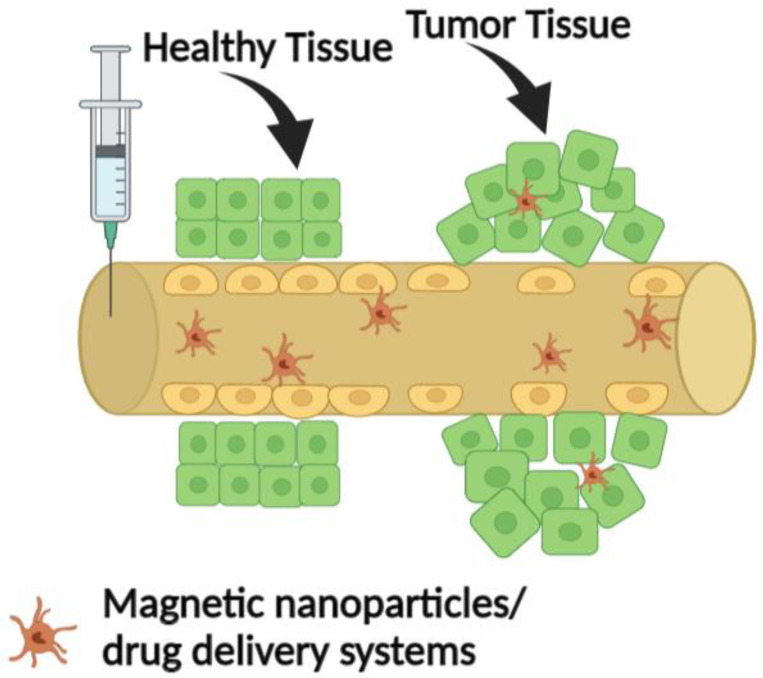
Passive targeting of tumour tissue occurs in blood vessel construction.

As is known from the literature, tumours develop a series of abnormal vessels through which the blood supply takes place, and abnormal vessels that present cracks through which NPs can accumulate in tumours [86,88]. As observed in Figure 3, there is an essential difference in the blood vessel construction between healthy and tumour tissue. As the tumoral tissue grows, the blood vessels are hastily created to keep pace with the increasing need for nutrients. This generates poorly constructed blood vessels permeable to the magnetic nanoparticles or other nanometre drug delivery systems. In addition, these nanoparticles are preferentially accumulated in the tumoral cells and tissues because more blood vessels irrigate these tissues, and their uptake is upregulated. By this passive targeting, therapeutics only target the tumour cells, leaving the healthy ones unharmed. 

## 6. Active Targeting

**Figure 4:**
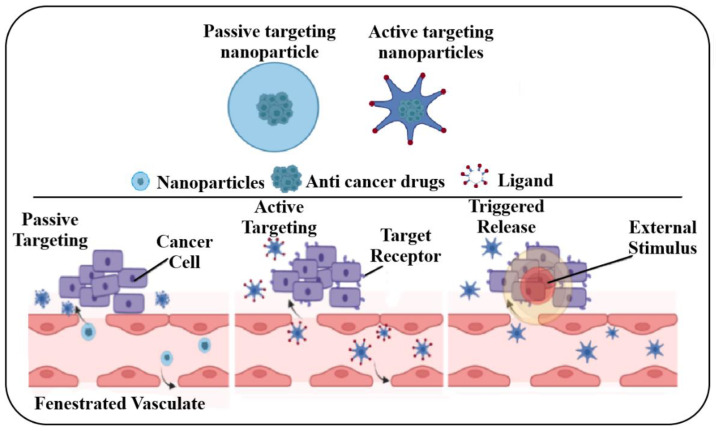
Action mechanism of passive and active targeting.

In treatments with magnetic support, biologically active molecules are conjugated with the surface of MNPs or could be encapsulated in the MNPs, such as liposomes, micelles, or dendrimers [85,89]. Thus, applying an external magnetic field to the charged MNPs with active molecules should be brought to and maintained in the area of interest. Figure 4 illustrates the action mechanism of passive and active targeting.

To understand the mechanism of action of passive and/or active targeting, one must comprehend the specifically targeted organ, and which method is appropriate for the best outcome. As already presented in the previous section, passive targeting is essential and correlated to the accumulation of MNPs and their circulation time. In contrast, active targeting occurs precisely, directly delivering therapeutics to the targeted cancerous cells by ligands to receptors [89,90].

## 7. Smart Magnetic Drug Delivery Systems 

**Figure 5:**
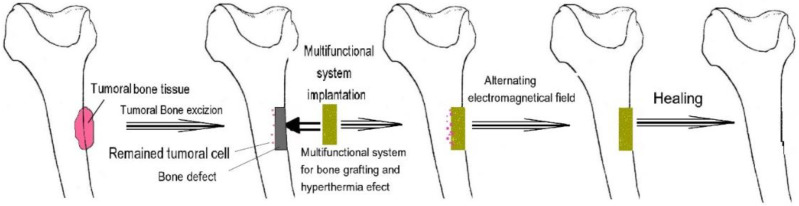
The procedure of treating osteosarcoma using multifunctional materials (with the kind permission of Springer [92]).

A simple, smart drug delivery system is represented by COLL/HA-Fe_3_O_4_@cisplatin [91], which was proposed to treat bone cancer. In this case, the multifunctional magnetic composite material loaded with cisplatin is implanted in the bone defect, as presented by Andronescu et al. [92,93]. In this case, such multifunctional materials can be used, and the loco-regional delivery is suitable to avoid systemic toxicity (Figure 5). These systems can be considered smart because they can be externally controlled. Due to the induced hyperthermia, the cisplatin delivery rate can be enhanced; thus, the antitumoral activity is enhanced. Such systems are suitable candidates for assuring personalized therapy, and depending on the evolution of the healing, the active agents’ release can be adapted.

**Figure 6:**
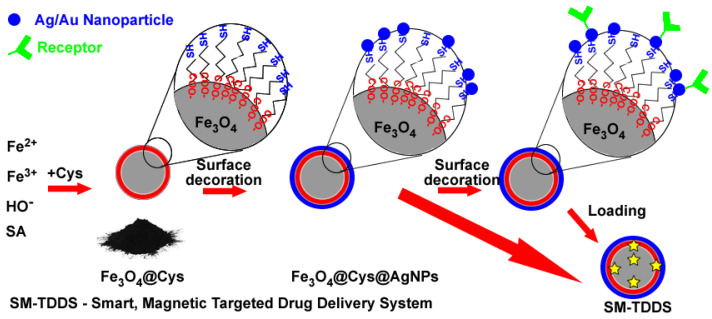
Smart MNP with triggered delivery and targeted capacity [96].

Due to its unique properties, such as high stability and the possibility of very easy functionalization via thiol (–SH) linkers [94], gold (and silver) is one of the most frequently used materials as a decorating material [95]. It is well known that various sulphur compounds, such as thiols and more, have a high affinity for gold [61]. Gold-coated MNPs were described in the literature in 2001, when researchers [68], using the reverse micellar mechanism, synthesized multifunctional systems of the “Fe_3_O_4_@Au” type with a core–shell structure and diameters 18–80 nm. These systems were later functionalized for binding biomolecules using -SH as linkers with an amine functional group [61]. When magnetic core and Au/Au nanoparticles are assembled via the thiolic linkers, the obtained systems can be used in cancer therapy. Magnetite nanoparticles can ensure magnetic targeting and produce hyperthermia. At the same time, these nanoparticles can exhibit photothermic effects and antimicrobial activity while providing the specific binding of receptors. If the silver content is high enough, core@shell@shell structures (Fe_3_O_4_@Cys@NM, where NM = noble metals, Au/Ag) can be obtained. The as-obtained magnetic systems can be loaded with adequate antitumoral drugs, and their release will be triggered by NIR or magnetic fields (Figure 6) [96].

Recently, several studies have developed silica based MNPs for cancer therapy which will be presented next. Hsiao et al. [97] developed an innovative theranostic platform consisting of L-cysteine-grafted mesoporous folic acid-europium-gadolinium-silica (FA-EuGd-MSNs-SS-Cys). This innovative platform has proven to be an effective tool for transporting, imaging, and delivering therapeutic drugs. The advantage of this approach is to function simultaneously as a therapeutic and guided ageing agent in cancer treatment. Another example showed that Fe_3_O_4_@SiO_2_@ tannic acid NPs are used as pH-sensitive drug delivery systems to simultaneously release the anticancer drugs methotrexate (MTX) and doxorubicin (DOX). Findings reported that Fe_3_O_4_@SiO_2_@Tann could be considered a double-shell nano-drug platform for treating cancer [98,99]. 

Dai et al. [100] developed a DOX-loaded Fe_3_O_4_@SiO_2_ platform by the solvothermal method. The results showed that 82.8% of lung cancer cells were killed by Fe_3_O_4_@SiO_2_@DOX treatment with a drug concentration of only 10 μg/mL of DOX. Furthermore, 81.3% of lung cancer cells were killed during incubation with Fe_3_O_4_@SiO_2_@DOX with a concentration of only 0.5 μg/mL of DOX and 15 min of NIR irradiation, thus proposing a unique synergism between chemotherapy and the photothermal effect [100]. 

Recently, combining chemotherapy and photothermal therapy with strong theranostic NPs, and integrating diagnostic and therapeutic agents has been a tremendously beneficial alternative in the fight against cancer. Elbialy et al. [101] developed multifunctional magnetic gold NPs (MGNPs) conjugated with PEG and DOX type MGNP_DOX. It was found that by integrating chemo/photothermal treatment, MGNP_DOX had higher efficiency both in vivo and in vitro. Additionally, examination of MGNP-DOX by immunohistochemical and histopathological studies confirmed using it as a theranostic material. Additionally, using MGNP-DOX as an MRI contrast agent for synergistic chemo/photothermal targeted therapies has led to promising results.

As acknowledged worldwide, triple-negative breast cancer (TNBC) is a very aggressive cancer cured via standard chemotherapy. Therefore, it is foremostly required to develop an innovative approach to treating TNBC [102]. In the presented study, Li et al. [103] fabricated a multifunctional magnetic gold hetero nanostructure with photosensitizer Ce_6_ (chlorine e6) loading (MF_MGN@Ce_6_) for synergic effects of photodynamic and photothermal (PDT/PTT) ability of TNBC. The obtained nano-drug MF_MGN@Ce_6_@RT was functionalized with mitochondria-targeting molecular and cell membrane-targeting peptide of cRGD of TPP. Results confirmed the guaranteed efficiency of the MF_MGN@Ce_6_@RT to TNBC tumours.

Lately, using natural polysaccharides in nanomedicine applications has gained significant interest. Chitosan (CS) has been used in cancer therapies as a drug delivery nanocarrier with promising achievements in targeted drug delivery [104,105]. Shanavas et al. [106] synthesized a hybrid based on MNPs with a core of PLGA functionalized with a folate-CS shell as an MRI contrast having anticancer features. The folate/CS shell was obtained from carbodiimide covered by SPIONs with a thin layer of PLGA loaded with docetaxel. In conclusion, it has been shown that the biocompatible hybrid core–shell has immense potential for simultaneous MR imaging and cancer treatment [106].

Adimoolam et al. [90] designed MNPs conjugated with DOX via a pH-sensitive imine bond with glutaraldehyde as a cross-linker. Cell viability tests confirmed that the MNPs conjugated DOX presented an improved therapeutic impact. An important outcome of MNPs was the low toxicity to the normal cell, which was assigned to the precise targeting capacity.

Recently, magnetic carbon-based nanomaterials gained significant attention due to their ability to develop platforms in which ligands or drugs are conjugated for cancer therapy applications. An example was Ag@Fe_3_O_4_@C_PEG_FA (FA-folate) NPs loaded with DOX, which have suitable biocompatibility and stability and exhibit synergistic potential and targeting capacity without any toxic side effects. Therefore, it has been proven that Ag@Fe_3_O_4_@C_PEG_FA NPs are suitable nano-platforms for chemo/photothermal therapy and imaging contrast [107]. 

Another example of a nano-drug platform for cancer therapy is discussed next. Kievit et al. [108] synthesized a new formulation based on chitosan-PEG-PEI coated on SPIONs functionalized with chlorotoxin (CTX) and green fluorescent protein (GFP) encoded DNA. The use of chlorotoxin is justified based on the targeting capacity of this ligand to brain tumours, such as glioma. After administering the complex DNA_CTX in the C_6_ xenograft tumour in mice, Kievit discovered an increased uptake of the complex DNA_CTX in the targeted tumour compared to the control DNA. Therefore, Kievit et al. [109] demonstrated the importance of copolymer coating of Fe_3_O_4_ NPs in targeting and gene therapy applications [110].

Even if there have been made significant breakthroughs in clinical trials with various nano-drug based on MNPs platforms towards cancer theranostic applications, further research still needs to be done to overcome many issues related to long-term toxicity and nano-bio human toxicity interactions. However, despite their importance in many clinical studies, especially in cancer therapy, no MNPs formulations are approved for therapeutic use [110]. Additionally, a significant problem was directing the target towards the organ, with each method presenting advantages and disadvantages.

Tumour delivery of macromolecular drugs based on MNPs has attracted great interest in cancer therapy. However, macromolecular drugs could be proteins, peptides, DNA-based constructs, and even lipids. It has been demonstrated that many proteins and peptides have enhanced biological effects, making them perfect therapeutics for developing anticancer agents. It is well known that tumour tissues differ anatomically from normal tissues, which makes them well-distinguished. Using macromolecules for cancer therapy has the advantage of penetrating and accumulating only in tumour tissues, which leads to extensive pharmacological accumulation. To achieve significant development and therapeutic potential of the anticancer drugs, they must attach macromolecules to based-targeting nanoparticles. One of the most commonly used nanoparticles as nanocarriers for targeted therapy are iron oxide nanoparticles, which are used as contrast agents for specific targeted drug delivery [54,111,112].

Yang et al. [113] developed an enzyme-responsive hybrid DOX-SMNPs (silica MNPs) for selective drug delivery and intracellular tumour imaging. The hybrid of enzyme-responsive nanoparticles demonstrated effective DOX release upon specific enzyme interaction in vitro. This multi-responsive hybrid exhibits better cellular tracking of DOX molecules through the help of MRI and fluorescence imaging techniques. 

Another study presented an anticancer drug delivery system based on MNPs grafted with carboxymethyl chitosan (CS) and β-cyclodextrin (β-CD). The obtained nano-drug was designed to enhance the delivery of prodigiosin (PG) to cancer cells. The anticancer drug, prodigiosin, loaded into the nanoparticles, was used as a model antitumor drug, targeting aggressive tumour cells. The results show that CS-MNPs presented efficiency and better targeting ability for prodigiosin toxicity effect on cancerous cells than β-CD-MNPs [114].

Wang et al. [115] synthesized polyethylenimine (PEI)-MNPs as nanocarriers for tumour treatment of glioblastoma multiforme, a very aggressive type of malignant brain tumour. Scientists are working to develop effective treatments for curing this bold, incurable brain tumour. The literature provides numerous intriguing insights regarding using surviving as a potential new target for cancerous tumour treatment. Currently, controlling the expression of surviving RNA might be the best approach for cancer research. Additionally, it is a valuable device for specific proteins encoded by mRNA, which become potential cancer therapeutic [116]. Therefore, choosing the appropriate carrier system for small interfering RNA (siRNA) delivery was challenging. MNPs are viable carriers for siRNA delivery due to their unique properties, such as size, nontoxicity, biocompatibility, great stability, and easy functionalization. Therefore, developing PEI-MNPs for siRNA gene delivery has practical applications in brain tumours. Results confirmed that fabricating PEI-MNPs with a cationic polymeric shell could effectively absorb adequate siRNA molecules and protect them from enzymatic media in vitro. This study indicated that the nanocarriers presented successful results in imaging and siRNA delivery for in vitro therapy of glioblastoma multiforme [115].

Another study developed a new magnetic nanovector targeting transgene therapy for oral squamous cell carcinoma (OSCC). The magnetic nanovector fabricated from MNPs modified by PEI polymer was tested as gene transfer vectors. The results indicated that PEI-modified Fe_3_O_4_ nanoparticles could target Tca83 cell killing and provide a potentially novel method for the future treatment of the OSCC [117].

Tanaka et al. [118] developed an intratumoral injection of immature dendritic cells (DCs) combined with magnetite cationic liposomes (MCL) with the induced effect of hyperthermia in vitro studies. When the DCs were pulsed with mouse B16 melanoma-heated cells, major histocompatibility complex (MHC) I/II and costimulatory molecules (CD80/CD86 and CCR7) were upregulated, concluding in DCs maturation. Additionally, it has been reported that DCs regulate the immune response in tumour cancer and have proven that they were triggered by heat shock proteins (HSPs). Very important to mention is that HSP70 induces antitumor immunity after hyperthermia. Therefore, suggesting that injecting DCs into tumour tissue will release HSP70 after hyperthermia implies that using magnetite nanoparticles is possible for malignant melanoma.

Another example of nanocarriers for cancer therapy was developed by Sun et al. [119], demonstrating that coupling DOX with BMs (bacterial magnetosomes) displayed efficient antitumour properties. In this study, bacterial magnetosomes (BMs) were used as carriers in cancer therapy and coupling DOX with BMs (DBMs) showed compatible results similar to DOX. BMs’ potential as drug carriers for enzymes, nucleic acids, and antibodies has been experimentally used for developing magnetic-targeted drug carriers. Results indicated that BMs showed good biocompatibility, and DBMs may offer an innovative target in cancer therapy; thus, clinical studies must be done to overcome any research obstacles.

Colon cancer is considered one of the most aggressive types of cancer. Therefore, studies have shown that the anticancer activity of human cathelicidin LL-37 peptide attached to the surface of MNPs would considerably be improved. Niemirowicz et al. [120] used two colon cancer culture cells (DLD-1 and HT-29 cells), LL-37 antimicrobial peptide and its synthetic analogue ceragenin CSA-13 (mimic peptides). They developed two novel nanosystems based on MNPs, MNP@LL-37 and MNP@CSA-13, to evaluate the effect of MNPs as a drug delivery system. Results proved that combining antimicrobial peptides with MNPs drug systems will decrease the viability of the colorectal cancer cell line. Additionally, studies showed that the ceragenin CSA-13 had enhanced apoptotic properties on colon cancer cells than LL-37. Hence, these results show that both nanosystems are excellent instruments in developing targeted therapy, mainly due to their capability to be incorporated into tumorous cells [121,122]. 

In another study, Gao et al. [123] developed new nanoplatforms for drug delivery based on nanomagnetic liposomes (Lips)- encapsulated parthenolide (PTL) and glucose oxidase (GOD) with efficient synergic antitumour therapy. This multifunctional drug was synthesized from modified MNPs, PTL and GOD-encapsulated to form the delivery system GOD-PTL-Lips@MNPs, targeting the tumour’s acidic environment. The formed nanoplatforms drug delivery system showed a significant antitumour effect, minimising the tissues’ toxicity in vivo. Through the synergistic effect of the constituent compounds of the drug delivery system, it has been provided with a possible approach, which could improve the efficiency in targeting and curing cancerous tumours. 

## 8. Conclusions

As cancer is a disease provoked by the uncontrolled division of the cells, any antitumor drug is a killing agent or is a substance that stops the natural multiplication of the cells. Therefore, being an anti-growing substance, it will affect both tumoral and healthy cells, hence the unwanted side effects of the chemotherapy. The new therapies are counting on target delivery of the drugs with the help of appropriate nanocarrier. As the drug is encapsulated, the new paradigm is to manage the delivery only to the tumour tissue. Magnetic nanocarriers present the distinct advantage of following an external magnetic field and concentrating in the tumour zone. In addition, these nanocarriers can generate hyperthermia and stress, which induces apoptosis and/or necrosis of the tumoral cells. However, maybe more importantly, the increased temperature can assure a triggered delivery. Thus, the active agent can be released at the desired moment and according to a desired rate.

Moreover, by decorating these nanoparticles with adequate molecules or applying an appropriate magnetic field, targeted accumulation and cell internalization can be achieved in the desired tumoral tissue/organ. Hyperthermia is controlled externally by using the correct electromagnetic field. The triggering and targeting can ensure an efficient therapy. At the same time, the release can be tuned according to the field characteristics and applied time to create the premises of personalized therapy/treatment.

## Data Availability

These data can be available upon request.
